# Supplementary material for: Identification of DAXX as a restriction factor of SARS-CoV-2 through a CRISPR/Cas9 screen
Source: Nat Commun. 2022 May 4;13:2442. doi: 10.1038/s41467-022-30134-9 (PMC9068693; doi:10.1038/s41467-022-30134-9)
Supplement: Supplementary file 1 — Supplementary Information [file 41467_2022_30134_MOESM1_ESM.pdf]

Supplementary information : Identification of DAXX As A Restriction Factor Of SARS-CoV-2 Through A CRISPR/Cas9 Screen

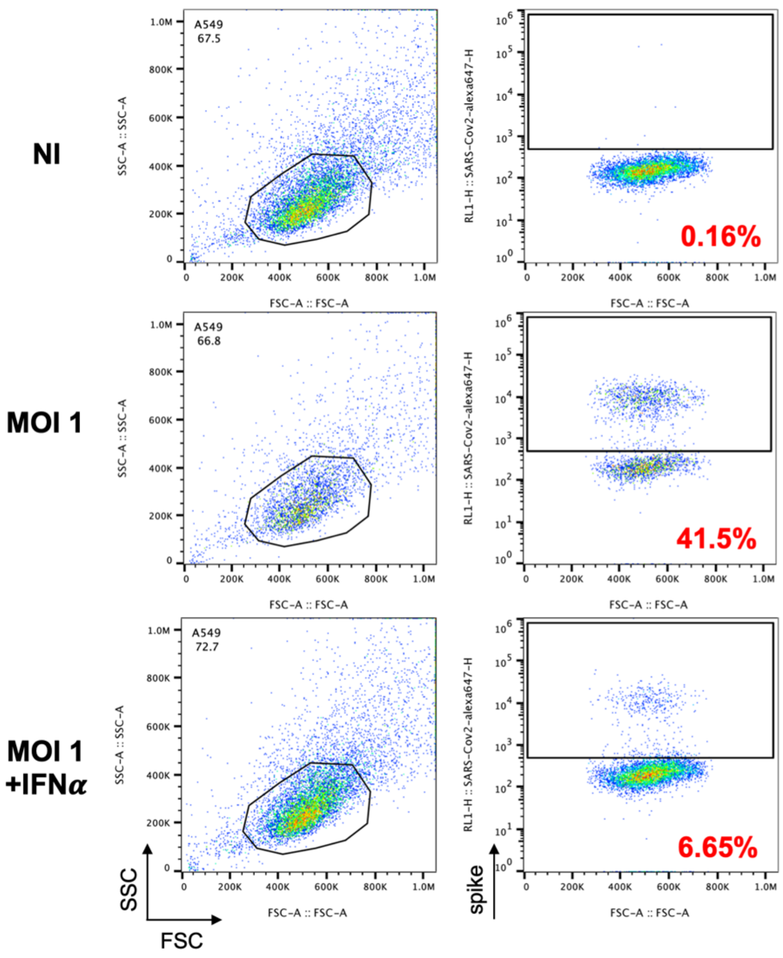

Supplementary Figure 1: IFN-mediated restriction of SARS-CoV-2. A549-ACE2 cells were pre-treated with 200 U/mL of IFNα and infected at MOI 1. Cells were labelled with anti-spike (S) antibody at 24h p.i. and the numbers of cells positive for S were analyzed by flow cytometry. Non-infected cells were used for gating controls. Percentage of infected cells are indicated in red. One representative experiment.

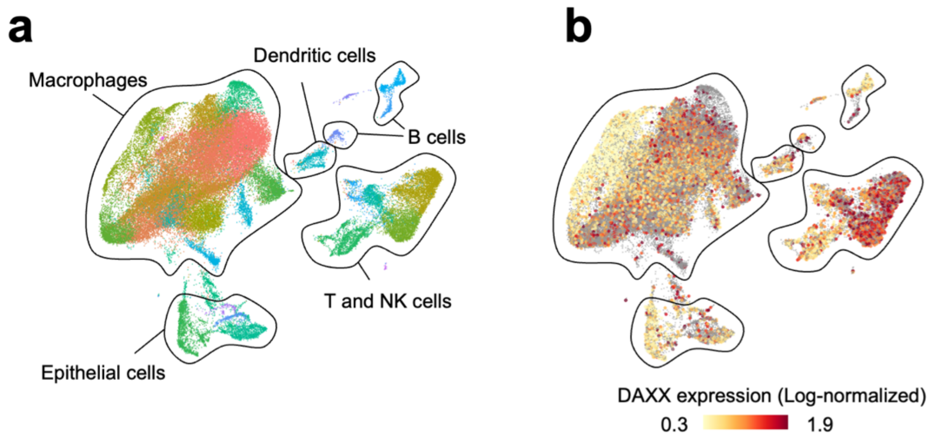

Supplementary Figure 2: Expression of DAXX RNA in cells isolated from broncho-alveolar lavages. a: Single cell RNAseq data of broncho-alveolar lavages from Liao *et al.* 2020 (43) (dataset ID: GSE145926) were analyzed using BBrowser Software. Colors indicate graph-based clusters. Cell types are indicated according to dataset metadata. Each dot represents an individual cell. **b:** Log-normalized expression of DAXX among single cells. Null values are excluded from the scale and indicated as grey dots.

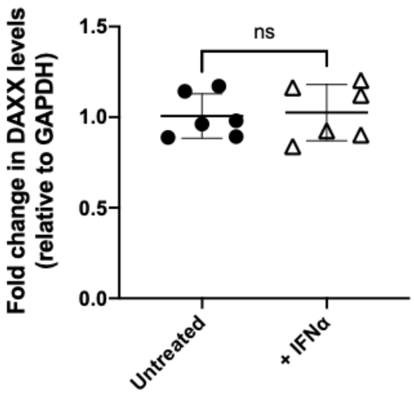

Supplementary Figure 3: DAXX expression following IFNα treatment. A549-ACE2 WT cells were treated with 200 U/mL of IFNα for 24h in triplicates. Cell monolayers were harvested. Cellular RNAs were extracted and DAXX levels were quantified by qRT-PCR analysis. qRT-PCR against the housekeeping gene GAPDH was used as a control and to normalize DAXX levels. The mean +/- SD of the fold changes in IFNα treated cells relative to control cells for 2 independent experiments is shown. Statistics: Unpaired two-sided t-test. ns: p-value > 0.05.

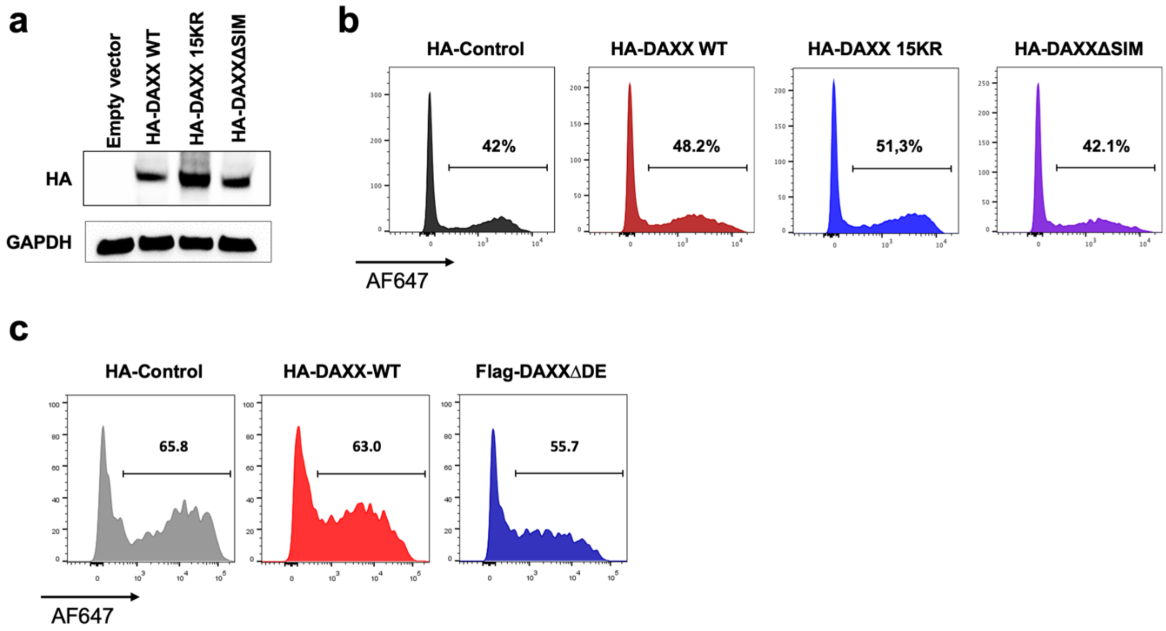

Supplementary Figure 4: Expression of WT DAXX and mutants in transfected 293T-ACE2 cells. 293T-ACE2 cells were transfected with the indicated HA-tagged DAXX constructs or with HA-NRB1 as a negative control. Levels of DAXX expression was measured by Western Blot (probing for HA; GAPDH as a loading control) in **a** and by flow cytometry (intracellular HA and Flag stainings) in **b-c.** One representative Western Blot out of 3 independent experiments is shown.

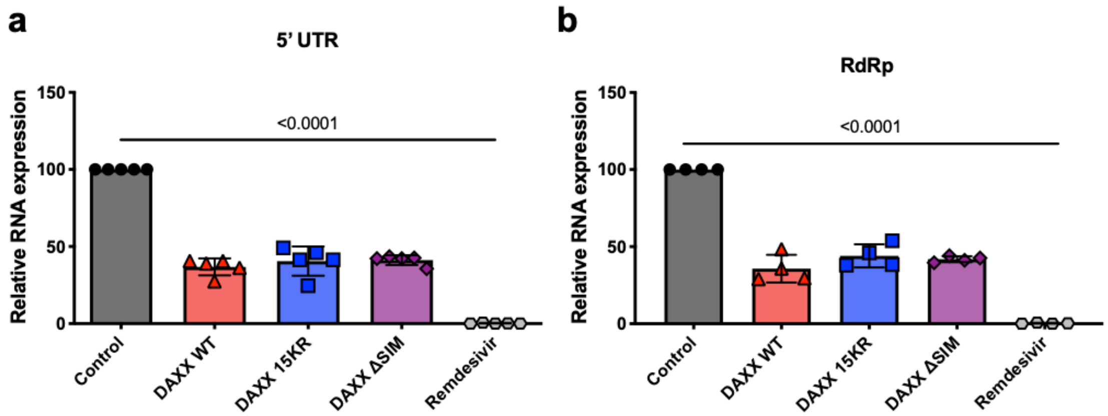

Supplementary Figure 5: Effect of DAXX overexpression on SARS-CoV-2 transcription. In parallel of the experiments shown in **Fig. 4b-c**, the intracellular levels of viral RNA (5' UTR ; RdRp) were quantified by qRT-PCR (normalized against RLP13a, ΔΔCt method). The mean of 4 independent experiments +/- SD is shown. Statistics: one-way ANOVA. P-values are indicated on the graph.

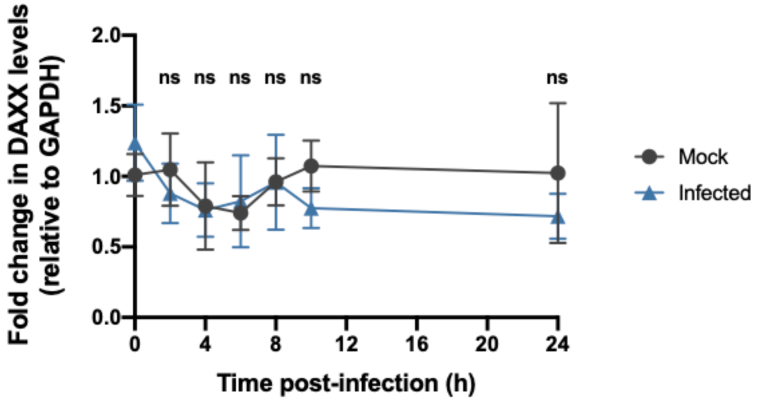

Supplementary Figure 6: DAXX mRNA levels are not affected by SARS-CoV-2 replication. A549-ACE2 WT cells were infected with SARS-CoV-2 at MOI 1. Cellular monolayers were harvested at the indicated time points and total RNA was extracted. The levels of DAXX RNA were determined by qRT-PCR analysis and normalized against GAPDH levels. The mean +/- SD of 3 independent experiments performed in triplicates is shown. Statistics: 2-way ANOVA using Sidak's test. ns: p-value > 0.05.

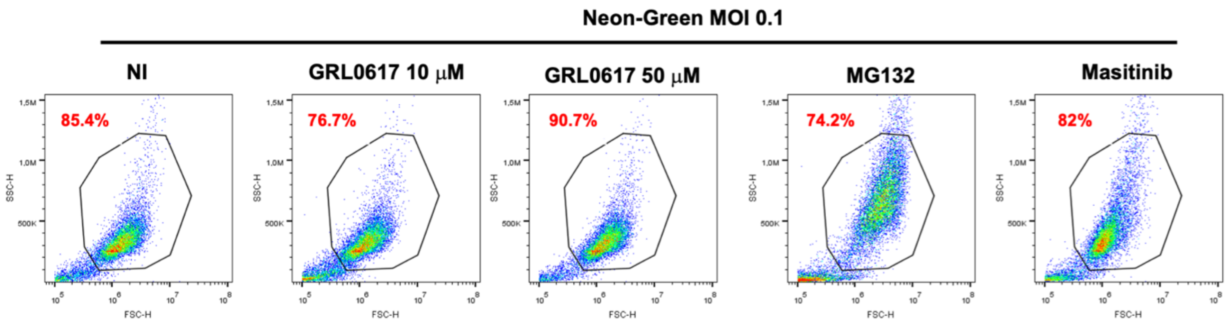

Supplementary Figure 7: FACS analyses of 293T-ACE2 treated with inhibitors.
The size (FSC) and granularity (SSC) of the cells used for the western-blots shown in **Fig. 6b** were evaluated by flow cytometry. The estimated percentage of live cells is indicated.

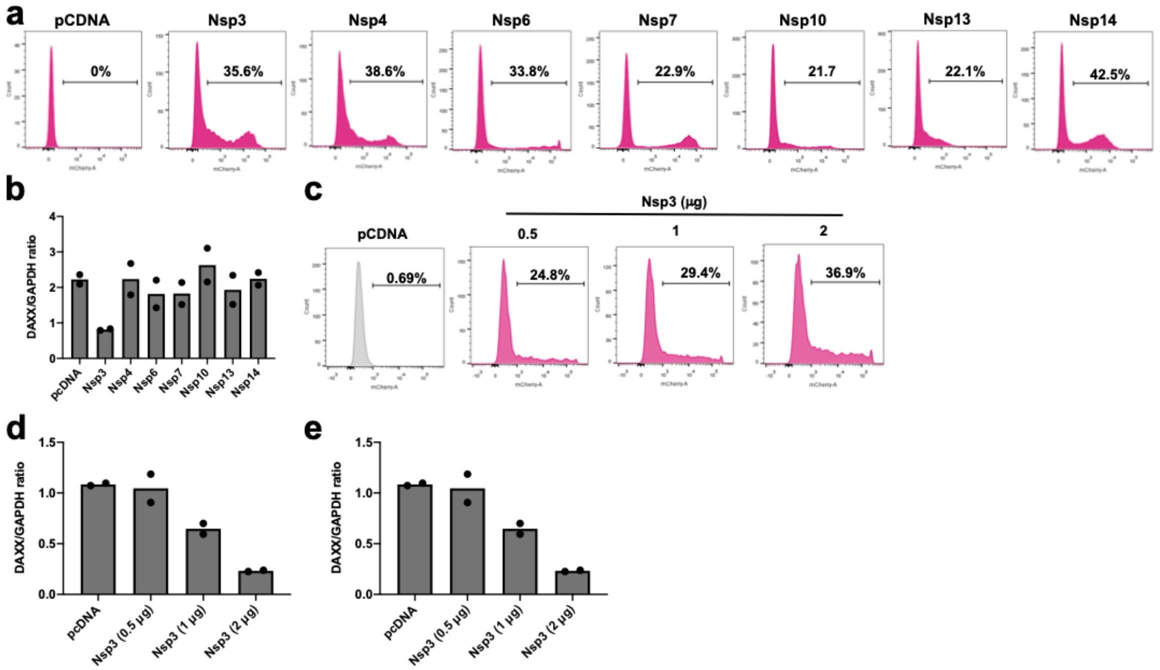

Supplementary Figure 8: Expression levels of Nsp-mCherry fusion proteins in 293T-ACE2 cells.

a: The expression of SARS-CoV-2 Nsp proteins fused to mCherry (**Fig. 6d**) was evaluated by flow cytometry. **b:** Quantification of Western Blot analyses from **Fig. 6d**. The intensity of the DAXX and GAPDH bands was determined using the Image Lab Software. The mean +/- SD of the DAXX/GAPDH ratio is represented (n=2). **c:** The expression of SARS-CoV-2 Nsp3-mCherry (**Fig. 6e**) was evaluated by flow cytometry. **d-e:** Quantification of Western Blot analyses from **Fig. 6e** is shown in **(d)** and of **Fig. 6f** in **(e)**. The intensity of the DAXX and GAPHD bands was determined using the Image Lab Software. The mean +/- SD of the DAXX/GAPDH ratio is represented (n=2).

Name	Sequence 5’ ⇒ 3’
PCR1 Forward Primer	GAGGGCCTATTTCCCATGATTCCTTCA
PCR1 Reverse Primer	aacttctcggggactgtgg
PCR2 Forward Primer 1 (non infected cells)	AATGATACGGCGACCACCGAGATCTACACTCTTTCCCTACACGACGCTCTTCC GATCTAGTCAAtcttgtggaaaggacgaaacaccg
PCR2 Forward Primer 2 (infected cells)	AATGATACGGCGACCACCGAGATCTACACTCTTTCCCTACACGACGCTCTTCC GATCTATGTCAtcttgtggaaaggacgaaacaccg
PCR2 Reverse Primer	CAAGCAGAAGACGGCATACGAGATGTGACTGGAGTTCAGACGTGTGCTCTTCC GATCTtgccactttttcaagttgataacggact

Supplementary Table 1 : NGS oligos for the CRISPR/Cas9 screen.

Gene	sgRNA1	sgRNA2	sgRNA3
LY6E	GGCCUGGCACUCACCAAUGC	GCCGACCAUCUGCUCCGACC	GGAGAAGCACAUCAGCGAGC
DAXX	CAGCACGAUGAUGCUGUUAG	CUCCCACCCACUCCCCAAUG	UCUGAGCCUCAUGGGGCCAG
APOL6	UCCAGAGAUGACAGCAGUAG	CAAUAUCGUCUGCGAGGGCA	CUCAAAAAUAUUUUUUCUUC
HERC5	CAACAACUGGGAGAGCCUUG	AGAAAAUUUCUAAAGCUUCU	CAGAUUAUCUUUGAGGCAGG
CTSL	UACUGUUGCCUCAUAUGGAU	AGGCUGCAAUGGUGGCCUAA	AGAUAAGCCUCCCAGUUUUC
IFI6	GAAAAAGUGCUCGGAGAGCU	ACCUCCUCCGACGGCCAUGA	CCUCCAGGACUCGCAGUCGC
IFNAR1	AAACACUUCUUCAUGGUAUG	GAGUGAAGAAAAGUUGCAUU	UUUACUUUAAAGAACUGGGA

Supplementary Table 2: sgRNAs sequences for KO pool generation.

Gene	Forward PCR primer	Reverse PCR primer	Sequencing primer
LY6E	CTGGCCACACTGTCTCACTG	TGCATGGGAAATGAGGCTGT	CTGTCTCACTGTGTGTTTGAGTGTC
DAXX	GGAACTAGAAGGTTCAGGGGA	TGGAGGGGCTCATTCTGAGG	GAACTAGAAGGTTCAGGGGAAGAAGGAAG
APOL6	TGTAGGGAGGTACAGGGAGG	TACCACTCACGATGCTGGTG	GATTCGAAGCTGAGAGTGGCAAGAATATC
HERC5	GGAGGCTAGGTGAGAAGGGA	GTCTTTCCACTGAGAAGACAGGT	GAAGGGATGTAAACAGGGGTTTTAGAAAAC
CTSL	GGTAGACTTTAAAGTGATGTACAGTTCA	ACCCCACCCAGCCCTAATAT	CAGTTCACTTTTTAACAGTATTCAGATGTG
IFI6	AGTAAAGAACGTCCCACCAGG	AAGTCCCTTCCCCTCTGTGA	AGTAAAGAACGTCCCACCAGG
IFNAR1	AGAGTGGAAGGGTGTATGCT	CTTGGAAAGTGAACTGCTCTG	TGCTAAAATGTTAATAGGACATTAGCTCAA

Supplementary Table 3: Primers used for sequencing of edited *loci.*

Targeted gene	Foward Primer	Reverse Primer	Probe
SARS-CoV-2 N	TAATCAGACAAGGAACTGATTA	CGAAGGTGTGACTTCCATG	
SARS-CoV-2 5' UTR	TGTCGTTGACAGGACACGAG	TTACCTTTCGGTCACACCCG	
SARS-CoV-2 RdRp	CATGTGTGGCGGTTCACTAT	TGCATTAACATTGGCCGTGA	
GAPDH	GAAGGTGAAGGTCGGAGTC	GAAGATGGTGATGGGATTTC	
DAXX	GGGCGACTATGTGAGCTGAA	GGCTTGTTGATGAGCCGCTC	
MERS-CoV E	GCAACGCGCGATTCAGTT	GCCTCTACACGGGACCCATA	CTCTTCACATAATCGCCCCGAGCTCG
SARS-CoV N	TGGACCCACAGATTCAACTGA	GCTGTGAACCAAGACGCAGTAT	TAACCAGAATGGAGGACGCAATGG
RPL13A	AACAGCTCATGAGGCTACGG	TGGGTCTTGAGGACCTCTGT	

Supplementary Table 4: qRT-PCR primers and probes.
