# Supplementary material for: Identification of DAXX as a restriction factor of SARS-CoV-2 through a CRISPR/Cas9 screen
Source: Nat Commun. 2022 May 4;13:2442. doi: 10.1038/s41467-022-30134-9 (PMC9068693; doi:10.1038/s41467-022-30134-9)
Supplement: Supplementary file 3 — Description Supplementary Data [file 41467_2022_30134_MOESM3_ESM.docx]

**Description of Additional Supplementary Information**

**Supplementary Data 1**

**Title :** sgRNA enrichment analysis

**Description :** Counts in the infected (treatment) and non-infected (control) cell populations are indicated for each sgRNA present in the library, together with log fold changes (LFC) and statistical analyses.

**Supplementary Data 2**

**Title :** MAGeCK analysis - gene score

**Description :** The MAGeCk algorithm assigns a score and p-value to each gene targeted in the library, based on the read counts presented in Supplementary Data 1. The full MAGeCK output is available at <https://github.com/Simon-LoriereLab/crispr_isg_sarscov2>.
