# Supplementary material for: Identification of DAXX as a restriction factor of SARS-CoV-2 through a CRISPR/Cas9 screen
Source: Nat Commun. 2022 May 4;13:2442. doi: 10.1038/s41467-022-30134-9 (PMC9068693; doi:10.1038/s41467-022-30134-9)
Supplement: Supplementary file 6 — Reporting Summary [file 41467_2022_30134_MOESM6_ESM.pdf]

nature research

Corresponding author(s): Ferdinand ROESCH, Marco VIGNUZZI, Nolwenn JOUVENET, Sébastien NISOLE

Last updated by author(s): Feb 15, 2022

Reporting Summary

Nature Research wishes to improve the reproducibility of the work that we publish. This form provides structure for consistency and transparency in reporting. For further information on Nature Research policies, see our Editorial Policies and the Editorial Policy Checklist.

Statistics

For all statistical analyses, confirm that the following items are present in the figure legend, table legend, main text, or Methods section.

n/a	Confirmed	
☐	☒	The exact sample size (*n*) for each experimental group/condition, given as a discrete number and unit of measurement
☐	☒	A statement on whether measurements were taken from distinct samples or whether the same sample was measured repeatedly
☐	☒	The statistical test(s) used AND whether they are one- or two-sided<br>*Only common tests should be described solely by name; describe more complex techniques in the Methods section.*
☒	☐	A description of all covariates tested
☐	☒	A description of any assumptions or corrections, such as tests of normality and adjustment for multiple comparisons
☐	☒	A full description of the statistical parameters including central tendency (e.g. means) or other basic estimates (e.g. regression coefficient) AND variation (e.g. standard deviation) or associated estimates of uncertainty (e.g. confidence intervals)
☐	☒	For null hypothesis testing, the test statistic (e.g. *F*, *t*, *r*) with confidence intervals, effect sizes, degrees of freedom and *P* value noted<br>*Give P values as exact values whenever suitable.*
☒	☐	For Bayesian analysis, information on the choice of priors and Markov chain Monte Carlo settings
☒	☐	For hierarchical and complex designs, identification of the appropriate level for tests and full reporting of outcomes
☒	☐	Estimates of effect sizes (e.g. Cohen's *d*, Pearson's *r*), indicating how they were calculated

Our web collection on statistics for biologists contains articles on many of the points above.

Software and code

Policy information about availability of computer code

Data collection	BD FACS DIVA v8 and Attune NxT Software v2.7 were used for flow cytometry data collection. QuantStudio v1.3 was used for RTqPCR data collection. Zen Black v8.1 was used for confocal microscopy data collection.
Data analysis	Statistical analyses: Graphpad Prism v9.1.0 was used to perform statistical analyses (1- and 2-way ANOVA, multiple comparisons, ...). RStudio v1.2.1335 was used to perform Dunett's tests on linear models and Holm correction of p-values.<br><br>CRISPR/Cas9 screen: Reads were demultiplexed using bcl2fastq Conversion Software v2.20 (Illumina) and fastx_toolkit v0.0.13 (http://hannonlab.cshl.edu/fastx_toolkit/). Sequencing adapters were removed using cutadapt v1.9.1 (https://cutadapt.readthedocs.io/en/v1.9.1/). The reference library was built using bowtie2 v2.2.9 (http://bowtie-bio.sourceforge.net/bowtie2/index.shtml). Read mapping was performed with bowtie2 allowing 1 seed mismatch in --local mode and samtools v1.9 (http://www.htslib.org/). Mapping analysis and gene selection were performed using MAGeCK v0.5.6 (https://sourceforge.net/projects/mageck/), normalizing the data with default parameters. MAGeCK score plot was built with R v3.6.3 (https://www.r-project.org/) using tidyverse (https://www.tidyverse.org/blog/2019/11/tidyverse-1-3-0/), ggrepel (https://github.com/slowkow/ggrepel), cowplot (https://www.rdocumentation.org/packages/cowplot/versions/1.1.1) packages.<br><br>Flow Cytometry.<br><br>FlowJo v10.8.1 was used for flow cytometry analysis.<br><br>Single cell analysis.<br><br>Single cell RNAseq analysis were performed in the BioTuring Browser Software v2.8.42

Image analysis

ImageJ (FiJi) v2.1 and Image lab v6 were used for image analysis.

For manuscripts utilizing custom algorithms or software that are central to the research but not yet described in published literature, software must be made available to editors and reviewers. We strongly encourage code deposition in a community repository (e.g. GitHub). See the Nature Research guidelines for submitting code & software for further information.

Data

Policy information about availability of data

All manuscripts must include a data availability statement. This statement should provide the following information, where applicable:

- Accession codes, unique identifiers, or web links for publicly available datasets
- A list of figures that have associated raw data
- A description of any restrictions on data availability

CRISPR/Cas9 screen NGS raw (fastq) and preprocessed data (read counts) is available in NCBI GEO (GSE173418, https://www-ncbi-nlm-nih-gov.ezproxy.u-pec.fr/geo/query/acc.cgi?acc=GSE173418); sgRNA and gene enrichment analyses are available in Supplementary Tables 5 and 6, respectively and full MAGeCK output at https://github.com/Simon-LoriereLab/crispr_isg_sarscov2.
The single cell data from Liao et al. 2020 Nature is available in NCBI GEO (GSE145926, https://www.ncbi.nlm.nih.gov/geo/query/acc.cgi?acc=GSE145926)
Source data are provided with this paper.

Field-specific reporting

Please select the one below that is the best fit for your research. If you are not sure, read the appropriate sections before making your selection.

- [x] Life sciences
- [ ] Behavioural & social sciences
- [ ] Ecological, evolutionary & environmental sciences

For a reference copy of the document with all sections, see nature.com/documents/nr-reporting-summary-flat.pdf

Life sciences study design

All studies must disclose on these points even when the disclosure is negative.

Sample size	No statistical estimation of sample size was performed. Sample size for each experiment was determined depending on practical limitations.
Data exclusions	Some individual values were excluded when the difference with the other 2 cognate replicates was too high. This was only done for RTqPCR experiments performed in triplicate, in which aberrant Ct values (>35 when other replicate values were <30) could occasionnaly be observed, likely indicating pipetting errors (e.g. sample was not added in the well).
Replication	All experiments were replicated independently at least twice and yielded consistent results. The costly NGS experiment was done once.
Randomization	Samples were not randomized across experiments. This is not necessary as the readout methods (RTqPCR; flow cytometry, confocal microscopy, etc) are unbiased and are not influenced by the order in which samples are processed.
Blinding	Blinding was not necessary in this study as no human-derived biological material was used and that readouts were unbiased (see above remark for randomization). Output of the NGS experiment was analyzed with an automated method. Analyses were performed in an unbiased manner, with pre-established criteria, and by different persons than those who collected and prepared the samples.

Reporting for specific materials, systems and methods

We require information from authors about some types of materials, experimental systems and methods used in many studies. Here, indicate whether each material, system or method listed is relevant to your study. If you are not sure if a list item applies to your research, read the appropriate section before selecting a response.

Materials & experimental systems

n/a	Involved in the study	
[ ]	[x]	Antibodies
[ ]	[x]	Eukaryotic cell lines
[x]	[ ]	Palaeontology and archaeology
[x]	[ ]	Animals and other organisms
[ ]	[x]	Human research participants
[x]	[ ]	Clinical data
[x]	[ ]	Dual use research of concern

Methods

n/a	Involved in the study	
[x]	[ ]	ChIP-seq
[ ]	[x]	Flow cytometry
[x]	[ ]	MRI-based neuroimaging

Antibodies

Antibodies used	Mouse anti-DAXX, Abnova MAB10621, clone 7A11, lot B1PM01040F00330<br>Rabbit anti-DAXX, Proteintech 20489-1-AP, lot 00013032<br>Rat anti-HA, Roche 11867423001, clone 3F10, lot 42155800<br>Mouse anti-dsRNA, Scicons 10010200, clone J2, lot J2-2106<br>Mouse anti-GAPDH, EMD Millipore MAB374, clone 6C5, lot 3273148<br>Goat anti-Lamin B clone M-20 ,lot A0813 (Santa Cruz sc-6217)<br>Mouse monoclonal HSP90α/β clone F-8, lot G2420 (Santa Cruz sc-13119)<br>Mouse monoclonal β-actin clone AC-15, lot 087M4850V (Sigma #A1978)<br>Mouse monoclonal α-Tubulin clone DMA1, lot 047M4789V (Sigma #T9026),<br>Rabbit anti-TRIM22, lot 13744-1-AP (Proteintech #13744-1-AP)<br>Mouse Monoclonal RIG-I clone Alme-1 (adipoGen #AG-20B-0009).<br>Mouse anti-spike clone 1A9, lot 44174 (GeneTex GTX632604)<br>Goat anti-mouse HRP-conjugate, lot 17061552 (GE Healthcare clone NA931V)<br>Goat anti-rabbit HRP-conjugate, lot 16963367 GE Healthcare NA934V,<br>Goat anti-rabbit AF555, lot 2272588 (Thermo Fisher A-21428)<br>Goat anti-mouse AF488, lot 2370705 (Thermo Fisher A-28175)<br>Goat anti-rat AF647, lot 2251195 (Thermo Fisher A-21247)<br>Goat anti-human AF647 Lot 2160390 (Invitrogen A21445)<br>Donkey anti-mouse AF647, lot 1757130 (Invitrogen A31571)<br>Mouse anti-S2 H2 162 antibody (gift from Dr. Hugo Mouquet, Institut Pasteur, Paris, France).
Validation	The Mouse anti-DAXX clone 7A11 antibody was validated by Abnova for Western Blot (K-562 cell lysate) and IF (against HeLa cells). Full information available at http://www.abnova.com/products/products_detail.asp?catalog_id=MAB10621<br>The Rabbit anti-DAXX clone 20489-1-AP antibody was validated by Proteintech for Western Blot (Y79 cells, HeLa cells) and IF (A431 cells). Full information available at https://www.ptglab.com/products/DAXX-Antibody-20489-1-AP.htm<br>The Rat anti-HA clone 3F10 antibody was validated by the Roche for Western Blot, IP, and ELISA. Full information available at https://www.sigmaaldrich.com/FR/fr/product/roche/roahaha?gclid=Cj0KCQiAu62QBhC7ARIsALXijXRnKCs0FyJ0QvEIij4-zrr8FI4BIGgY34Itil45nRqsFJ0ShySSswcaAuuhEALw_wcB<br>The Mouse anti-dsRNA clone J2 antibody was validated by Scicons for ELISA, Flow Cytometry, Immunocytochemistry. Full information available at https://zageno.de/p/anti-dsrna-mab-j2/4760896#resources<br>The Mouse anti-GAPDH clone 6C5 antibody was validated by EMD Millipore for WB (A431 lysates). Full information available at https://www.merckmillipore.com/FR/fr/product/Anti-Glyceraldehyde-3-Phosphate-Dehydrogenase-Antibody-clone-6C5,MM_NF-MAB374#anchor_TI<br>The Goat anti-Lamin B clone M-20 was validated by Santa Cruz for Western Blot. Full information available at https://www.scbt.com/p/lamin-b-antibody-m-20<br>The Mouse monoclonal HSP90α/β clone F-8 antibody was validated by Santa Cruz for Western Blot using IMR-32, Jurkat, NIH/3T3 and KNRK whole cell lysates Full information available at https://www.scbt.com/p/hsp-90alpha-beta-antibody-f-8<br>The Mouse monoclonal β-actin clone AC-15 antibody was validated by Sigma for Western Blot using rat liver protein lysates. Full information available at https://www.sigmaaldrich.com/FR/fr/product/sigma/a1978<br>The Mouse monoclonal α-Tubulin clone DMA1 antibody was validated by Sigma for Western Blot. Full information available at https://www.sigmaaldrich.com/US/en/product/sigma/t9026<br>The Rabbit anti-TRIM22 was validated by Proteintech for Western Blot using mouse thymus tissue. Full information available at https://www.ptglab.com/products/TRIM22-Antibody-13744-1-AP.htm<br>The Mouse Monoclonal RIG-I clone Alme-1 antibody was validated by adipoGen for Western Blot using HeLa cells lysates. Full information available at https://adipogen.com/ag-20b-0009-anti-rig-i-mab-alme-1.html/<br>The Mouse anti-spike clone 1A9 antibody was validated by GeneTex for IF using BHK-21 cells transfected with full-length SARS-CoV-2 spike. Full information available at https://www.genetex.com/Product/Detail/SARS-CoV-SARS-CoV-2-COVID-19-spike-antibody-1A9/GTX632604<br>The Mouse anti-S2 H2 162 antibody was validated by the team of Hugo Mouquet (Institut Pasteur, Paris, France) using SARS-CoV-2 infected Vero and A549-ACE2 cells.

Eukaryotic cell lines

Policy information about cell lines

Cell line source(s)	HEK 293T (ATCC #CRL-11268), VeroE6 (ATCC #CRL-1586) and A549 (ATCC #CCL-185) were obtained through ATCC. 293T and A549 overexpressing ACE2 were generated in the lab of Olivier Schwartz. A549-ACE2 KO cell lines were generated by Synthego Corporation.
Authentication	HEK 293T, VeroE6 and A549 cells were authenticated by ATC by STR profiling. The engineered A549 cells (ACE2 WT and KO) were authentificated by morphology; flow cytometry ACE2 stainings; and by Sanger sequencing of the CRISPR/Cas9 edited loci.
Mycoplasma contamination	All cell lines tested negative for mycoplasma.
Commonly misidentified lines<br>(See ICLAC register)	No cross-contaminations were reported according to ICLAC for the cell lines used in this study

Human research participants

Policy information about studies involving human research participants

Population characteristics	We only used the single cell data published in the Liao et al. 2020 Nature study, and did not perform experiments with human participants ourselves. This study indicates that "the median age was 57 years, and the participants included six male and three female patients. All nine patients had Wuhan exposure history and had cough and/or fever as the first symptom. Diagnosis of SARS-CoV-2 was based on clinical symptoms, exposure history, chest radiography and were SARS-CoV-2 RNA-positive using commercial quantitative PCR with reverse transcription (qRT–PCR) assays in the sputum, nasal swab and/or BALF. Influenza A and B virus infection were excluded at enrollment."
Recruitment	The Liao et al 2020 Nature study indicates that "all 13 patients with COVID-19 were enrolled from the Shenzhen Third People's Hospital from January to February, 2020. Disease severity was defined as moderate, severe and critical, according to the 'Diagnosis and Treatment Protocol of COVID-19 (the 7th Tentative Version)' by the National Health Commission of China issued on 3 March 2020 (http://www.nhc.gov.cn/yzygj/s7653p/202003/46c9294a7dfe4cef80dc7f5912eb1989.shtml). "
Ethics oversight	The Liao et al. 2020 Nature study indicates that "this study was conducted according to the principles expressed in the Declaration of Helsinki. Ethical approval was obtained from the Research Ethics Committee of Shenzhen Third People's Hospital (2020-112). All participants provided written informed consent for sample collection and subsequent analyses."

Note that full information on the approval of the study protocol must also be provided in the manuscript.

Flow Cytometry

Plots

Confirm that:

- [x] The axis labels state the marker and fluorochrome used (e.g. CD4-FITC).
- [x] The axis scales are clearly visible. Include numbers along axes only for bottom left plot of group (a 'group' is an analysis of identical markers).
- [x] All plots are contour plots with outliers or pseudocolor plots.
- [x] A numerical value for number of cells or percentage (with statistics) is provided.

Methodology

Sample preparation	For the cell sorting experiment, A549-ACE2 cells were harvested by trypsin treatment and fixed for 15 min in Formalin 1%. Fixed cells were washed in cold FACS buffer containing PBS, 2% Bovine Serum Albumin (Sigma-Aldrich #A2153-100G), 2 mM EDTA (Invitrogen #15575-038) and 0.1% Saponin (Sigma-Aldrich #S7900-100G).<br><br>For overexpression experiments, 293T-ACE2 cells were harvested from tissue culture plates by trypin treatment and fixed with 4% formaldehyde. For intracellular staining, cells were permeabilized in a in a PBS 1% BSA 0.025% saponin solution for 30 min prior to staining with corresponding antibodies for 1h at 4°C diluted in the permeabilization solution.
Instrument	For cell sorting, cells were sorted on a Aria Fusion Cytometer (Becton Dickinson). For flow cytometry analysis, cells were analyzed on a Fortessa (Becton Dickinson) or on an Attune NxT cytometer.
Software	Data were collected on FACSDiva v8 or Attune NxT v2.7 and analyzed using FlowJo v10.8.1 (Treestar Inc., Oregon, USA).
Cell population abundance	1.9x10^6 infected cells were recovered post-sorting. Cells were sorted with the "4-way purity" setting but not re-analyzed post-sorting.
Gating strategy	For the cell sorting experiment, live cells were gated on FSC-A/SSC-A basis. DIVA doublet discrimination gating was used to remove cell doublets. Spike-A647 positive cells were gated as indicated in Fig.1A.<br><br>For overexpression experiments, live cells were gated on FSC-A/SSC-A basis and then on HA-AF647-positive cells. Percentage of infected (dsRNA+) within this population is provided on all plots shown in Fig. 3C.

- [x] Tick this box to confirm that a figure exemplifying the gating strategy is provided in the Supplementary Information.
